# Supplementary material for: Clinicopathological Features of Kidney Injury Related to Immune Checkpoint Inhibitors: A Systematic Review
Source: J Clin Med. 2023 Feb 8;12(4):1349. doi: 10.3390/jcm12041349 (PMC9964206; doi:10.3390/jcm12041349)
Supplement: Supplementary file 1 [file jcm-12-01349-s001.zip › jcm-2125193-supplementary.pdf]

Supplementary materials

Supplemental Table S1. PubMed search strategy.

Supplemental Table S2. Definition of kidney outcomes of AKD and non-AKD patients

Supplemental Table S3. Summary of study characteristics, safety and efficacy of all included case report and case series.

Supplemental Table S4. Summary of cohort studies.

Supplemental Table S5. The Newcastle-Ottawa scale (NOS) quality assessment of the enrolled studies.

Supplemental Table S6. Quality assessment of case/case series.

Supplemental Table S7. Risk factors for ICI-associated kidney IRAEs with ATIN/AIN and ATN

Supplemental Table S8. Risk factors for ICI-associated kidney IRAEs with glomerular disease and systematic disease

Supplemental Table S9. Univariate and multivariable logistic analysis of factors with kidney function recovery in AKD patients

Supplemental Table S10. Univariate and multivariable logistic analysis of factors with tumor progression and death

Supplemental Table S1. PubMed search strategy.

# No	Searches
Part I: Immune checkpoint inhibitor	
**1**	"Programmed Cell Death 1 Receptor"[MeSH Terms] OR "CTLA-4 Antigen"[MeSH Terms]
**2**	"anti-PD-1"[Title/Abstract] OR "PD-1"[Title/Abstract] OR "anti-PD-L1"[Title/Abstract] OR "PD-L1"[Title/Abstract] OR "anti pd l 1"[Title/Abstract] OR "pd l 1"[Title/Abstract] OR "CTLA-4"[Title/Abstract] OR "anti-CTLA-4"[Title/Abstract] OR "anti-cytotoxic T-lymphocyte antigen-4"[Title/Abstract]
**3**	"nivolumab"[Title/Abstract] OR "pembrolizumab"[Title/Abstract] OR "atezolizumab"[Title/Abstract] OR "durvalumab"[Title/Abstract] OR "avelumab"[Title/Abstract] OR "ipilimumab"[Title/Abstract] OR "cemiplimab"[Title/Abstract]
**4**	"immune checkpoint inhibitor"[Title/Abstract] OR "immune checkpoint inhibitors"[Title/Abstract] OR "ICI"[Title/Abstract] OR "immune checkpoint blockade"[Title/Abstract] OR "ICB"[Title/Abstract])
**5**	1 or 2 or 3 or 4
Part II: Renal adverse events	
**6**	("Acute kidney injury"[All Fields] OR "Nephritis"[Title/Abstract] OR "Amyloidosis"[All Fields] OR "acute tubular necrosis"[Title/Abstract] OR "minimal change disease"[Title/Abstract] OR "Immunoglobulin A nephropathy"[Title/Abstract] OR "Glomerulopathy"[Title/Abstract] OR "acute tubulointerstitial nephritis"[Title/Abstract]
**7**	"nephrotoxicit*"[Title/Abstract] OR "kidney toxicit*"[Title/Abstract] OR "kidney injur*"[Title/Abstract] OR "renal injur*"[Title/Abstract] OR "renal insufficienc*"[Title/Abstract] OR "kidney insufficienc*"[Title/Abstract] OR "kidney failure*"[Title/Abstract] OR "renal failure*"[Title/Abstract]
**8**	6 or 7
Part III : Part I AND II	
**9**	5 and 8

* means wildcard symbol, For example, "injur*" means the search would find all the words starting with “injur” including injury, injuries, injured and so on.

Supplemental Table S2. Definition of kidney outcomes of AKD and non-AKD patients

Kidney outcomes of AKD patients	
Complete recovery	last Scr decreased to the baseline[a] or higher than baseline within 26.5 µmol/L
Partial recovery	last Scr decreased by 25% or more from peak Scr[b] but remaining higher than baseline over 26.5µmol/L
No recovery	last Scr[c] decreased by less than 25% from peak Scr or continue RRT
Kidney outcomes of non-AKD patients	
Proteinuria remission	≥50% reduction from peak proteinuria according to 24h urine protein quantification

Abbreviations: AKD, acute kidney disease; Scr, serum creatine; RRT, renal replacement treatment.

a. Baseline SCr was defined as the lowest SCr value before the occurrence of kidney IREAs.

b. Peak SCr was defined as the highest value reported in the original articles

c. Last SCr was defined as the last SCr value available in the original articles.

Supplemental Table S3. Summary of study characteristics, safety and efficacy of all included case report, case series and conference abstracts.

Study ID		Sex	Age	Cancer type	ICI regimen	Pathological type	AKD	AKD stage	Kidney recovery	Tumor response	Rechallenge	Recurrent irAE
Author	Year											
Okamoto, M.	2020	male	61	other	PD-1	MN	no	NA	NA	NA	NA	NA
Mamlouk, O.	2020	male	71	melanoma	PD-1+CTLA-4	MCD, IgA nephropathy	yes	1	yes	complete control	no	NA
Narayanankutty, N. P.	2019	female	77	lung cancer	PD-1	AIN	yes	3	yes	NA	no	yes
Charmetant, X.	2019	male	59	lung cancer	PD-1	AIN	no	NA	NA	NA	NA	NA
Marvania, N.	2020	male	70	other	PD-1	AIN	yes	3	yes	NA	NA	NA
Patel, V.	2020	male	71	renal cancer	PD-1	AIN	yes	1	yes	complete control	NA	NA
Patel, V.	2020	male	70	renal cancer	PD-1	AIN	yes	3	yes	partial control	no	NA
Patel, V.	2020	male	58	renal cancer	PD-1	IgA nephropathy	yes	3	yes	partial control	no	NA
Bottlaender, Lea	2017	female	76	melanoma	PD-1	AIN	yes	2	yes	partial control	yes	yes
Buyansky, Dimitry	2017	female	62	other	PD-L1	ATIN	yes	3	yes	NA	NA	NA
Mulroy, M.	2021	female	79	melanoma	PD-1	AIN	yes	3	yes	complete control	no	NA
Oleas, Diana	2021	female	59	lung cancer	PD-L1+CTLA-4	AIN	yes	3	yes	progressive disease	NA	NA
Oleas, Diana	2021	female	67	other	PD-L1	AIN	yes	3	yes	progressive disease	NA	NA
Oleas, Diana	2021	female	83	melanoma	PD-1	AIN	yes	3	yes	complete control	NA	NA
Oleas, Diana	2021	male	85	lung cancer	PD-1	AIN	yes	1	yes	progressive disease	NA	NA
Oleas, Diana	2021	female	68	lung cancer	PD-1	AIN	yes	2	yes	stable	NA	NA
Oleas, Diana	2021	male	63	lung cancer	PD-L1	AIN	yes	3	no	partial control	NA	NA

Supplemental Table S3. (continued)

Oleas, Diana	2021	male	75	melanoma	PD-1	AIN	yes	3	yes	complete control	NA	NA
Oleas, Diana	2021	male	61	lung cancer	PD-1	AIN	yes	3	yes	progressive disease	NA	NA
Georgianos, P. I.	2019	male	60	lung cancer	PD-1	AIN	yes	3	yes	NA	no	NA
Belliere, J.	2016	female	67	lung cancer	PD-1	AIN	yes	3	yes	NA	NA	NA
Belliere, J.	2016	female	52	melanoma	PD-1	AIN	yes	2	yes	NA	NA	NA
Belliere, J.	2016	female	68	melanoma	CTLA-4	AIN	yes	1	yes	NA	NA	NA
Ryuzaki, M.	2019	male	76	other	PD-1	ATIN	yes	3	yes	NA	no	NA
Ryuzaki, M.	2019	female	76	lung cancer	PD-1	AIN	yes	2	yes	NA	NA	NA
Meraz-Muñoz, A.	2020	male	55	melanoma	NA	TMA	yes	1	NA	NA	NA	NA
Meraz-Muñoz, A.	2020	female	56	melanoma	NA	TMA	yes	3	NA	NA	NA	NA
Meraz-Muñoz, A.	2020	female	63	melanoma	NA	AIN	yes	1	NA	NA	NA	NA
Meraz-Muñoz, A.	2020	male	68	melanoma	NA	AIN	yes	3	NA	NA	NA	NA
Meraz-Muñoz, A.	2020	male	69	melanoma	NA	AIN	yes	2	NA	NA	NA	NA
Meraz-Muñoz, A.	2020	male	76	melanoma	NA	AIN	yes	1	NA	NA	NA	NA
Meraz-Muñoz, A.	2020	male	66	melanoma	NA	AIN	yes	3	NA	NA	NA	NA
Meraz-Muñoz, A.	2020	male	42	hematologic cancer	NA	ATN, MCD	yes	3	NA	NA	NA	NA
Meraz-Muñoz, A.	2020	male	72	other	NA	AIN	yes	1	NA	NA	NA	NA
Meraz-Muñoz, A.	2020	male	45	melanoma	NA	MCD	no	NA	NA	NA	NA	NA
Meraz-Muñoz, A.	2020	female	53	other	NA	MN	no	NA	NA	NA	NA	NA
Meraz-Muñoz, A.	2020	male	39	other	NA	MN	no	NA	NA	NA	NA	NA

Supplemental Table S3. (continued)

Catapano, F.	2021	female	52	melanoma	PD-1	ATIN	yes	NA	yes	NA	NA	NA
Gordon, L.	2019	male	67	melanoma	PD-1+CTLA-4	AIN	yes	3	yes	NA	no	NA
Panthofer, A. M.	2020	female	73	melanoma	PD-1	TMA, Acute antibody mediated rejection	yes	3	no	NA	yes	NA
Irifuku, T.	2020	male	59	renal cancer	PD-1	ATIN, GN	yes	2	yes	stable	no	NA
Xipell, M.	2018	male	54	renal cancer	PD-L1	ATIN	yes	3	yes	partial control	no	NA
Basnet, S.	2019	female	62	lung cancer	PD-1	ATIN	yes	3	yes	progressive disease	no	NA
Uner, M.	2021	male	65	other	PD-1	AASV	yes	3	yes	NA	no	NA
Jolly, E. C.	2009	male	41	melanoma	CTLA4	AIN	yes	3	yes	progressive disease	no	NA
Sammartino, C.	2010	male	50	melanoma	CTLA-4	anti-GBM disease	yes	3	no	NA	no	NA
Noto-Kadou-Kaza, B.	2021	female	71	renal cancer	PD-1	AIN	yes	3	yes	NA	NA	NA
Shirali, A. C.	2016	male	73	lung cancer	PD-1	AIN	yes	1	yes	progressive disease	no	NA
Shirali, A. C.	2017	male	78	lung cancer	PD-1	AIN	yes	2	yes	NA	NA	NA
Shirali, A. C.	2018	female	60	lung cancer	PD-1	AIN	yes	3	yes	stable	no	NA
Shirali, A. C.	2019	female	69	lung cancer	PD-1	AIN	yes	2	yes	NA	no	NA
Shirali, A. C.	2020	male	59	lung cancer	PD-1+CTLA-4	AIN	yes	3	yes	NA	no	NA
Shirali, A. C.	2021	female	69	lung cancer	PD-1	AIN	yes	2	yes	NA	NA	NA
Izzedine, H.	2014	male	70	renal cancer	PD-L1	TMA	yes	1	yes	NA	no	NA
Izzedine, H.	2014	male	72	renal cancer	PD-L1	TMA	yes	1	no	NA	no	NA

Supplemental Table S3. (continued)

Martínez Valenzuela, L.	2021	male	70	lung cancer	PD-1	ATIN	yes	3	yes	complete control	no	NA
Martínez Valenzuela, L.	2021	male	80	melanoma	PD-1	ATIN	yes	3	yes	NA	no	NA
Martínez Valenzuela, L.	2021	female	60	renal cancer	PD-1	ATIN	yes	3	yes	progressive disease	no	NA
Chemaly, E. R.	2020	female	26	hematologic cancer	PD-1	AIN, MCD	yes	2	yes	progressive disease	no	NA
Chua, J.	2020	male	77	other	PD-1	ATIN	yes	3	no	NA	NA	NA
George, S.	2019	male	27	other	PD-1+CTLA-4	GN	yes	3	yes	NA	NA	NA
Mamlouk, O.	2020	male	40	lung cancer	PD-1	FSGS	yes	3	yes	progressive disease	NA	NA
Mamlouk, O.	2020	male	70	renal cancer	PD-1	FSGS	yes	2	yes	progressive disease	no	NA
Mamlouk, O.	2020	female	60	melanoma	PD-1+CTLA-4	vasculitis	yes	3	yes	NA	NA	NA
Mamlouk, O.	2020	male	60	other	PD-1	ATN, FSGS	yes	3	yes	NA	NA	NA
Mamlouk, O.	2020	female	50	melanoma	PD-1+CTLA-4	vasculitis	yes	3	yes	stable	no	NA
Mamlouk, O.	2019	male	40	melanoma	PD-1+CTLA-4	Acute antibody mediated rejection	yes	3	no	NA	yes	NA
Aratani, S.	2021	male	78	lung cancer	PD-1	ATIN	yes	3	yes	NA	no	NA
Aratani, S.	2021	male	73	lung cancer	PD-1	ATIN	yes	2	yes	NA	no	NA
Aratani, S.	2021	male	66	lung cancer	PD-1	AIN	yes	3	yes	NA	no	NA
Aratani, S.	2021	male	52	lung cancer	PD-1	ATIN	yes	1	yes	NA	no	NA
Cortazar, F. B.	2016	male	70	melanoma	CTLA-4	AIN	yes	3	yes	NA	yes	no

Supplemental Table S3. (continued)

Cortazar, F. B.	2016	male	64	melanoma	PD-1+CTLA-4	AIN	yes	3	yes	NA	no	NA
Cortazar, F. B.	2016	male	74	melanoma	PD-1+CTLA-4	AIN	yes	3	yes	NA	no	NA
Cortazar, F. B.	2016	female	62	melanoma	CTLA-4	AIN	yes	3	no	NA	no	NA
Cortazar, F. B.	2016	female	71	lung cancer	PD-1+CTLA-4	AIN	yes	3	yes	NA	no	NA
Cortazar, F. B.	2016	male	64	other	CTLA-4	AIN	yes	3	no	NA	no	NA
Cortazar, F. B.	2016	male	71	melanoma	PD-1	AIN	yes	3	yes	NA	yes	yes
Cortazar, F. B.	2016	male	58	melanoma	CTLA-4	TMA	yes	3	no	NA	no	NA
Cortazar, F. B.	2016	male	75	melanoma	PD-1+CTLA-4	AIN	yes	3	yes	NA	no	NA
Cortazar, F. B.	2016	female	32	hematologic cancer	CTLA-4	AIN	yes	2	no	NA	yes	no
Cortazar, F. B.	2016	female	73	melanoma	CTLA-4	AIN	yes	3	yes	NA	no	NA
Cortazar, F. B.	2016	male	66	other	PD-1	AIN	yes	3	yes	NA	no	NA
Cortazar, F. B.	2016	female	41	melanoma	PD-1	AIN	yes	2	yes	NA	no	NA
Pampols, P. A.	2020	male	70	lung cancer	PD-1	ATIN	yes	3	yes	NA	NA	NA
Pampols, P. A.	2020	male	82	melanoma	PD-1	ATIN	yes	3	yes	NA	NA	NA
Pampols, P. A.	2020	female	63	renal cancer	PD-1	ATIN	yes	3	yes	NA	NA	NA
Abramson, M.	2020	male	67	lung cancer	PD-1	crescentic GN	yes	3	yes	stable	no	NA
Gebauer, E.	2021	male	72	melanoma	PD-1+CTLA-4	ATIN	yes	3	yes	progressive disease	no	NA
Sise, M. E.	2019	male	60	melanoma	PD-1	AIN	yes	3	yes	stable	yes	yes
Lo, W. K.	2020	female	85	other	PD-1+CTLA-4	GN	yes	2	yes	NA	no	NA
Lo, W. K.	2020	male	67	other	PD-1+CTLA-4	GN	yes	2	yes	NA	no	NA

Supplemental Table S3. (continued)

Lo, W. K.	2020	male	74	renal cancer	PD-1	AIN, GN	yes	3	yes	NA	no	NA
Takahashi, N.	2018	male	74	lung cancer	PD-1	anti-GBM disease	yes	3	no	stable	NA	NA
Sebastian Hultin	2020	male	NA	melanoma	PD-1+CTLA-4	anti-GBM disease	yes	NA	NA	complete control	NA	NA
Sebastian Hultin	2020	male	NA	melanoma	PD-1+CTLA-4	ATIN	yes	NA	NA	NA	NA	NA
Sebastian Hultin	2020	male	NA	melanoma	PD-1+CTLA-4	ATIN	yes	NA	NA	NA	NA	NA
Sebastian Hultin	2020	male	NA	melanoma	PD-1+CTLA-4	ATIN	yes	NA	NA	NA	NA	NA
Sebastian Hultin	2020	male	NA	melanoma	PD-1+CTLA-4	ATIN	yes	NA	NA	NA	NA	NA
Sebastian Hultin	2020	male	NA	melanoma	PD-1	ATIN	yes	NA	NA	NA	NA	NA
Sebastian Hultin	2020	male	NA	melanoma	PD-1+CTLA-4	ATIN	yes	NA	NA	NA	NA	NA
Sebastian Hultin	2020	male	NA	melanoma	PD-1+CTLA-4	ATIN	yes	NA	NA	NA	NA	NA
Sebastian Hultin	2020	male	NA	melanoma	PD-1+CTLA-4	ATIN	yes	NA	NA	NA	NA	NA
Sebastian Hultin	2020	female	NA	melanoma	PD-1+CTLA-4	ATIN	yes	NA	NA	NA	NA	NA
Sebastian Hultin	2020	male	NA	melanoma	PD-1+CTLA-4	ATIN	yes	NA	NA	NA	NA	NA
Sebastian Hultin	2020	male	NA	melanoma	PD-1	ATIN	yes	NA	NA	NA	NA	NA
Tawhari, I.	2020	male	67	melanoma	PD-1+CTLA-4	ATIN	yes	3	yes	NA	no	NA
Leuprecht, L.	2020	male	62	other	PD-1+CTLA-4	AIN, IgA nephropathy	yes	3	yes	NA	NA	NA
Bobart, S.	2020	male	87	other	PD-1	vasculitis	yes	3	yes	NA	no	NA
Mohan, S.	2018	male	67	melanoma	PD-1+CTLA-4	vasculitis	yes	3	yes	NA	NA	NA
Savedchuk, S.	2019	male	67	renal cancer	PD-1	AIN	yes	3	yes	NA	NA	NA
Koda, R.	2018	male	67	lung cancer	PD-1	ATIN	yes	2	yes	stable	no	NA
Tadros, M. G.	2019	female	70	lung cancer	PD-1	AIN	yes	3	yes	NA	NA	NA
Montanez, M.	2020	female	52	lung cancer	PD-1	AASV	yes	3	yes	complete control	NA	NA

Supplemental Table S3. (continued)

Lin, J.	2020	male	62	melanoma	PD-1+CTLA-4	ATIN	yes	3	yes	NA	NA	NA
Lapman, S.	2020	male	42	other	PD-1	ATN, AA	yes	1	no	complete control	NA	NA
Lapman, S.	2020	male	75	other	PD-1	AA	no	NA	NA	partial control	NA	NA
Lapman, S.	2020	male	81	melanoma	PD-1	AA	yes	3	no	complete control	NA	NA
Duque, J.	2018	male	52	other	PD-1+CTLA4	AIN	yes	NA	yes	NA	NA	NA
Baradhi, K. M.	2018	female	82	lung cancer	PD-1	ATIN	yes	NA	yes	NA	NA	NA
Leger, K.	2019	male	49	renal cancer	PD-1	GN	yes	1	no	progressive disease	no	NA
Parza, K.	2021	male	73	melanoma	PD-1+CTLA-4	ATIN	yes	3	yes	NA	no	NA
Lin, J. S.	2021	female	69	other	PD-L1	ATIN	yes	3	yes	NA	NA	NA
Lin, J. S.	2021	male	67	melanoma	PD-1+CTLA-4	ATIN	yes	3	yes	partial control	NA	NA
Lin, J. S.	2021	male	76	melanoma	PD-1+CTLA-4	ATIN	yes	2	yes	complete control	NA	NA
Lin, J. S.	2021	female	64	other	PD-1	ATIN	yes	3	yes	stable	NA	NA
Lin, J. S.	2021	male	72	other	PD-L1	ATN	yes	3	yes	progressive disease	NA	NA
Lin, J. S.	2021	male	77	lung cancer	PD-1	ATN	yes	2	yes	progressive disease	yes	yes
Lin, J. S.	2021	male	77	other	PD-1	ATIN	yes	3	yes	progressive disease	NA	NA
Lin, J. S.	2021	female	52	lung cancer	PD-1	ATIN	yes	1	no	stable	no	NA
de Sousa Oliveira, D.	2019	female	19	hematologic cancer	PD-1	AIN	no	NA	NA	partial control	yes	no

Supplemental Table S3. (continued)

Oliveira, D. S.	2019	female	19	hematologic cancer	PD-1	AIN	no	NA	NA	progressive disease	yes	no
Escandon, J.	2017	male	64	melanoma	PD-1	ATIN	yes	3	yes	progressive disease	yes	no
Escandon, J.	2017	female	78	melanoma	PD-1	ATIN	yes	3	yes	NA	no	NA
Nagaraju, G.	2018	female	54	melanoma	PD-1	AIN	yes	NA	yes	complete control	no	NA
Thajudeen, B.	2015	male	74	melanoma	CTLA-4	AIN	yes	2	yes	NA	yes	no
Kidd, J. M.	2016	male	55	melanoma	CTLA-4	MCD	yes	3	yes	NA	NA	NA
Lemoine, M.	2019	male	70	melanoma	CTLA-4	vasculitis	yes	3	yes	progressive disease	no	NA
Izzedine, H.	2021	male	78	melanoma	CTLA-4	AIN	yes	3	yes	partial control	NA	NA
Izzedine, H.	2021	female	60	melanoma	CTLA-4	AIN	yes	NA	yes	NA	NA	NA
Cruz-Whitley, J.	2020	female	75	hematologic cancer	PD-1	crescentic GN	yes	1	yes	progressive disease	no	NA
Jensen, C.	2019	male	20	other	PD-1+CTLA-4	MN	no	NA	NA	NA	NA	NA
Meraz-Munoz, A. Y.	2019	female	52	other	PD-1	MN	no	NA	NA	NA	yes	yes
Meraz-Munoz, A. Y.	2019	male	39	other	PD-1	MN	no	NA	NA	NA	NA	NA
Gao, B.	2018	male	40	hematologic cancer	PD-L1	MCD	no	NA	NA	NA	no	NA
Toda, M. G.	2021	male	75	lung cancer	PD-1	MCD	no	NA	NA	NA	NA	NA
Vaughan, E.	2020	male	57	other	PD-1	MCD	yes	2	no	progressive disease	no	NA

Supplemental Table S3. (continued)

Makati, D.	2018	female	66	melanoma	PD-1+CTLA-4	AIN	yes	3	yes	NA	no	NA
Messias, Ana	2019	male	52	melanoma	PD-1	ATIN	yes	3	no	progressive disease	no	NA
Kitchlu, A.	2017	male	43	hematologic cancer	PD-1	MCD	yes	3	yes	progressive disease	no	NA
Kitchlu, A.	2017	male	45	melanoma	CTLA-4	MCD	no	NA	NA	progressive disease	yes	yes
Omar Mamlouk	2019	male	75	renal cancer	PD-1	ATIN, AASV	yes	2	NA	stable	NA	NA
Omar Mamlouk	2019	female	69	melanoma	PD-1+CTLA-4	vasculitis	yes	3	NA	stable	NA	NA
Omar Mamlouk	2019	male	69	melanoma	PD-1+CTLA-4	ATIN, IgA nephropathy	yes	1	NA	stable	NA	NA
Omar Mamlouk	2019	female	50	melanoma	PD-1	FSGS, IgA nephropathy	yes	3	NA	progressive disease	NA	NA
Omar Mamlouk	2019	female	60	renal cancer	PD-1	MN	yes	NA	NA	progressive disease	NA	NA
Omar Mamlouk	2019	female	61	hematologic cancer	PD-1	ATIN, GN	yes	3	NA	stable	NA	NA
Omar Mamlouk	2019	male	74	hematologic cancer	PD-1	ATIN, FSGS	yes	1	NA	progressive disease	NA	NA
Omar Mamlouk	2019	male	63	other	PD-1	ATN, AA	yes	3	NA	progressive disease	NA	NA
Omar Mamlouk	2019	male	41	lung cancer	PD-1	vasculitis	yes	3	NA	progressive disease	NA	NA

Supplemental Table S3. (continued)

Omar Mamlouk	2019	male	74	other	PD-1	AIN	yes	3	yes	partial control	NA	NA
Omar Mamlouk	2019	male	77	other	PD-1	ATIN	yes	3	yes	stable	NA	NA
Omar Mamlouk	2019	male	58	lung cancer	PD-1	ATN	yes	3	yes	stable	NA	NA
Omar Mamlouk	2019	male	65	other	PD-1	ATN	yes	3	NA	progressive disease	NA	NA
Omar Mamlouk	2019	male	68	melanoma	PD-1	ATIN	yes	3	NA	stable	NA	NA
Omar Mamlouk	2019	male	55	other	PD-L1	ATIN	yes	1	NA	progressive disease	NA	NA
Jung, Kyungsuk	2016	male	70	renal cancer	PD-1	ATN, GN	yes	3	yes	partial control	no	NA
Ai, Luoyan	2021	male	72	other	PD-1	AIN	yes	3	yes	complete control	no	NA
Shim, J.	2021	male	68	other	PD-1	GN	yes	2	yes	progressive disease	yes	no
Daanen, R. A.	2017	male	62	renal cancer	PD-1	FSGS	yes	3	yes	progressive disease	no	NA
Nakatani, Y.	2018	female	68	other	PD-1	ATIN	yes	3	yes	NA	yes	no
Tanabe, K.	2020	male	78	other	PD-1	IgA nephropathy	yes	2	no	progressive disease	no	NA
Wakabayashi, K.	2021	male	69	lung cancer	PD-1	MN	no	NA	NA	stable	no	NA
Hninn, W. Y.	2019	male	65	other	PD-1	AASV	yes	3	yes	NA	NA	NA
Leonard, D.	2015	male	63	melanoma	PD-1	AIN	yes	3	no	NA	no	NA
Karo, N. L.	2020	female	64	lung cancer	PD-1	AIN	yes	3	yes	NA	yes	NA
Annamaraju, P.	2020	female	51	lung cancer	PD-1	AASV	yes	2	no	NA	NA	NA

Supplemental Table S3. (continued)

Mukherjee, A.	2020	female	73	lung cancer	PD-1	ATIN	yes	3	yes	NA	NA	NA
Hassanein, M.	2020	female	81	lung cancer	PD-1	ATIN	yes	3	yes	NA	no	NA
Bickel, A.	2016	male	62	other	PD-1	MCD	yes	NA	yes	stable	no	NA
Hayata, Manabu	2020	male	68	other	PD-1	TMA	yes	3	no	NA	no	NA
Kim, D. W.	2021	female	46	melanoma	PD-1	FSGS	no	NA	NA	NA	no	NA
Padala, S. A.	2021	female	46	other	PD-1	Acute antibody mediated rejection	yes	3	no	partial control	no	NA
Bonilla, M.	2021	male	88	renal cancer	PD-L1	MN	yes	1	yes	progressive disease	no	NA
Venkatachalam, K.	2020	male	69	other	PD-1	Acute antibody mediated rejection	yes	3	no	progressive disease	NA	NA
Venkatachalam, K.	2020	male	68	melanoma	PD-1+CTLA-4	Acute antibody mediated rejection	yes	3	no	complete control	NA	NA
Thomas, M. J. A.	2018	female	73	melanoma	PD-1	AASV	yes	3	yes	progressive disease	NA	NA
Okawa, S.	2020	male	63	lung cancer	PD-1	ATIN	yes	3	yes	NA	NA	NA
Efe, O.	2020	female	52	lung cancer	PD-1	ATIN, GN	yes	3	yes	NA	no	NA
Faieta, A.	2021	male	65	renal cancer	PD-1	ATIN	yes	3	yes	stable	no	NA
Hu, Q.	2020	male	66	other	PD-1	ATN, IgA nephropathy	yes	2	yes	stable	yes	no
M. Espi	2020	male	66	lung cancer	PD-1	ATIN	yes	1	no	NA	NA	NA
M. Espi	2020	male	68	melanoma	PD-1	ATIN	yes	1	no	NA	NA	NA
M. Espi	2020	female	77	melanoma	PD-1	ATIN	yes	2	yes	NA	NA	NA

Supplemental Table S3. (continued)

M. Espi	2020	male	85	melanoma	PD-1	ATIN	yes	2	no	NA	NA	NA
M. Espi	2020	female	85	melanoma	PD-1	ATIN	yes	3	yes	NA	NA	NA
Oki, R.	2020	female	75	lung cancer	PD-1	ATIN, IgA nephropathy	no	NA	NA	progressive disease	no	NA
Rashidi, A.	2021	male	76	hematologic cancer	PD-1	ATIN	yes	2	yes	NA	NA	NA
Herrmann, S.	2021	male	60	lung cancer	PD-1	ATIN	yes	1	yes	NA	no	NA
Herrmann, S.	2021	male	70	lung cancer	PD-1	ATIN	yes	3	yes	NA	no	NA
Gallan, A. J.	2019	male	68	melanoma	PD-1	vasculitis	yes	1	no	NA	no	NA
Gallan, A. J.	2019	female	71	lung cancer	PD-1	AASV	no	NA	NA	NA	NA	NA
Gallan, A. J.	2019	female	75	lung cancer	PD-1	AIN	yes	3	yes	NA	NA	NA
Gallan, A. J.	2019	female	63	melanoma	PD-1	AIN, vasculitis	yes	3	yes	NA	NA	NA
Glutsch, V.	2019	male	68	melanoma	PD-1	MCD	yes	3	yes	progressive disease	yes	no
Chang, Y.	2020	male	72	other	PD-1	AIN	yes	2	yes	NA	NA	NA
Kawakado, K.	2021	male	63	lung cancer	PD-1	ATN	yes	3	yes	NA	NA	NA
Sebastian, A. D.	2018	male	75	lung cancer	PD-1	AA	no	NA	NA	partial control	NA	NA
Murakami, N.	2016	male	75	melanoma	PD-1+CTLA-4	AIN	yes	3	yes	NA	no	NA
Person, F.	2020	male	55	melanoma	PD-1+CTLA-4	AIN, TMA	yes	3	no	progressive disease	yes	no
Vishwanath, M.	2018	male	58	renal cancer	PD-1	Acute antibody mediated rejection	yes	3	no	NA	no	NA

Supplemental Table S3. (continued)

Molteni, A.	2018	female	69	melanoma	PD-1	ATIN	yes	NA	yes	NA	no	NA
Taki, T.	2020	male	75	lung cancer	PD-1	ATIN	yes	2	yes	progressive disease	yes	no
Saly, D. L.	2018	male	61	lung cancer	PD-1	ATIN	yes	3	yes	partial control	yes	yes
Tabei, A.	2018	male	57	lung cancer	PD-1	ATIN	yes	3	yes	NA	NA	NA
Pichler Sekulic, Simona	2020	male	61	lung cancer	PD-1	ATN	yes	3	yes	NA	NA	NA
Soellradl, I.	2018	male	58	melanoma	CTLA-4	Acute antibody mediated rejection	yes	NA	no	NA	yes	yes
Vakil, V.	2018	male	66	other	PD-1	ATIN	yes	1	yes	NA	NA	NA
Uchida, A.	2017	male	67	lung cancer	PD-1	ATIN	yes	3	yes	stable	no	NA
Shah, N.	2019	male	70	melanoma	PD-1	ATIN	yes	NA	yes	stable	NA	NA
Fadel, N.	2009	male	64	melanoma	CTLA-4	lupus nephritis	no	NA	NA	stable	no	NA

Abbreviations: AKD: acute kidney disease; irAE: immune-related adverse events; NA: not applicable; ATIN: acute tubulo-interstitial nephritis; ATN: acute tubular necrosis; AIN: acute interstitial nephritis; MCD: minimal change disease; FSGS: focal segmental glomerulosclerosis; MN: membranous nephropathy; GN: glomerulonephritis; AASV: ANCA-associated small vessel vasculitis; TMA: thrombotic microangiopathy; AA: renal amyloidosis

Supplemental Table S4. Summary of cohort studies.

	Alexandre O (n=63)	Hassan Izzedine (n=12)	Frank B. Cortazar (n=60)	Clarissa Cassol (n=15)	Juliana B(N=13)	Total (n=163)
**Age**	NA	69.75	64(58-73)	60.8±10.5	71±8.5	NA
**Male [n/N(%)]**	44/63	7/12	39/60	10/15	8/13	108/163
**Hypertension [n/N(%)]**	33/63	5/12	30/60	NA	7/13	75/148
**Diabetics [n/N(%)]**	4/63	1/12	9/60	NA	3/13	17/148
**CKD [n/N(%)]**	NA	NA	19/60	NA	NA	19/60
**CHD [n/N(%)]**	5/63	NA	NA	NA	NA	5/63
**Tumor type**						
**Melanoma [n/N(%)]**	24/63	9/12	20/60	7/15	3/13	63/163
**Lung cancer [n/N(%)]**	26/63	0/12	15/60	4/15	9/13	54/163
**Renal cancer [n/N(%)]**	5/63	0/12	NA	3/15	0/13	8/103
**Hematologic cancer [n/N(%)]**	1/63	1/12	NA	0/15	0/13	2/103
**Others [n/N(%)]**	7/63	2/12	NA	1/15	1/13	11/103
**ICI type**						
**PD-1/PD-L1 [n/N(%)]**	54/63	12/12	41/60	11/15	11/13	129/163
**CTLA-4 [n/N(%)]**	5/63	0/12	1/60	0/15	0/13	6/163
**Combination [n/N(%)]**	4/63	0/12	18/60	4/15	2/13	28/163
**Other treatment**						

Supplemental Table S4. (continued)

**Only ICI [n/N(%)]**	59/63	9/12	NA	13/14	13/13	94/102
**ICI+ targeted therapy [n/N(%)]**	0/63	1/12	NA	0/14	0/13	1/102
**ICI+ Chemotherapy [n/N(%)]**	2/63	2/12	NA	1/14	0/13	5/102
**ICI+VEGF [n/N(%)]**	2/63	0/12	NA	0/14	0/13	2/102
**Interval time (d)**	105.5±98.6	270(30-720)	NA	219±210	NA	NA
**PPI [n/N(%)]**	23/53	NA	33/60	6/11	9/13	71/137
**NSAIDs [n/N(%)]**	5/53	NA	NA	1/12	3/13	9/78
**Baseline Scr (mg/dl)**	1.09±0.26	NA	0.94(0.80-1.34)	3.2±1.4	1.01±0.32	NA
**Peak Scr (mg/dl)**	3.26±1.56	NA	NA	NA	4.19±1.43	NA
**AKD [n/N(%)]**	63/63	11/12	60/60	15/15	13/13	162/163
**AKD grade**						
**1 grade [n/N(%)]**	NA	NA	NA	NA	NA	NA
**2 grade [n/N(%)]**	NA	NA	NA	NA	NA	NA
**3 grade [n/N(%)]**	23/63	NA	NA	NA	NA	23/63
**Only Proteinuria [n/N(%)]**	0/63	1/12	0/60	0/15	0/13	1/163
**Extra renal irAE [n/N(%)]**	25/63	NA	22/56	6/15	NA	53/134
**Skin [n/N(%)]**	7/63	NA	6/56	1/15	NA	14/134
**Gastrointestinal [n/N(%)]**	8/63	NA	NA	1/15	NA	9/78
**Endocrine [n/N(%)]**	2/63	NA	NA	3/15	NA	5/78

Supplemental Table S4. (continued)

**Pneumonitis [n/N(%)]**	2/63	NA	1/56	0/15	NA	3/134
**Others [d] [n/N(%)]**	6/63	NA	NA	1/15	NA	1/78
**ATIN/AIN**	52/63	4/12	56/60	9/15	13/13	134/163
**ATN**	6/63	6/12	0/60	6/15	0/13	18/163
**MCD**	0/63	2/12	1/60	0/15	0/13	3/163
**Anti-GBM disease**	0/63	0/12	1/60	0/15	0/13	1/163
**Vasculitis**	1/63	0/12	1/60	0/15	0/13	2/163
**FSGS**	1/63	0/12	0/60	0/15	0/13	1/163
**Unkown GN**	2/63	0/12	0/60	0/15	0/13	2/163
**C3 GN**	0/63	0/12	1/60	0/15	0/13	1/163
**MN**	1/63	0/12	0/60	0/15	0/13	1/163
**Corticosteroid**	56/63	7/12	53/60	9/15	13/13	138/163
**Intravenous corticosteroid**	NA	NA	15/60	0/15	3/13	18/88
**Immunosuppressants**	0/63	0/12	7/60	NA	0/13	7/148
**Discontinued ICI**	63/63	10/12	60/60	6/10	NA	139/145
**RRT**	5/63	1/12	NA	1/15	NA	7/90
**Disruption of RRT**	NA	0/1	NA	1/1	NA	1/2
**Renal function recovery**						
**Complete recovery**	17/59	NA	26/60	7/11	6/13	56/143
**Partial recovery**	34/59	NA	25/60	NA	NA	59/119
**No recovery**	8/59	NA	9/60	NA	NA	17/119

Supplemental Table S4. (continued)

**Death**	1/63	2/12	NA	5/15	NA	8/90
**Rechallenge**	17/57	1/12	NA	0/15	NA	18/84
**Flare**	0/17	1/1	NA	0/0	NA	1/18

Abbreviations: HTN: hypertension; DM: diabetes mellitus; RRT: renal replacement therapy; IV-glucocorticoid: intravenous glucocorticoid; ATN: acute tubular necrosis; ATIN: acute tubulo-interstitial nephritis; AIN: acute interstitial nephritis; MCD: minimal change disease; FSGS: focal segmental glomerulosclerosis; TMA: thrombotic microangiopathy; MN: membranous nephropathy; GN: glomerulonephritis; AA: amyloidosis protein A; Anti-GBM disease: anti-glomerular basement membrane disease

Supplemental Table S5. The Newcastle-Ottawa scale (NOS) quality assessment of the enrolled studies.

Study ID	SELECTION				COMPARABILITY	OUTCOME			Total[a]
	Representativeness of the exposed cohort	Selection of the nonexposed cohort	Ascertainment of exposure	Demonstration that outcome of interest was not present at start of study[b]	Comparability of cohorts on the basis of the design or analysis[c]	Assessment of outcome	Was follow-up long enough for outcomes to occur[d]	Adequacy of follow up of cohorts[e]	
Juliana B. Draibe 2020	somewhat*	same institute*	record*	no	*	record*	yes*	yes*	7
Cassol, C. 2019	somewhat*	same institute*	record*	no	-	record*	yes*	yes*	6
Dinh, A. 2020	somewhat *	different source	record*	no	-	record*	yes*	yes*	5
Alexandre O. Gérard 2021	somewhat *	same institute*	record*	no	-	record*	yes*	yes*	6
Hassan Izzedine 2019	somewhat *	same institute*	record*	no	-	record*	yes*	yes*	6

- indicates Zero score, * indicates one score, ** indicates two scores

a. Each study could be awarded a maximum of nine stars: a maximum of two stars for the item regarding comparability and a maximum of one star for other 7 items

b. One score was awarded if a study was a prospective cohort study

c. A maximum of two stars could be awarded for this item. If a study performed landmark analysis, one score was awarded. If a study adjusted for confounding factors (eg. ECOG PS, age, metastases status, serum low density lipoprotein level, prior treatment line, etc.), an additional score was awarded

d. For studies reporting renal outcomes, one score was awarded.

e. If a study reported a follow up rate of more than or equal to 80%, one score was awarded

Supplemental Table S6. Quality assessment of case/case series/conference abstracts.

Study ID		Selection	Ascertainment		Causality				Reporting	Total Assessment
**Author**	**Year**	**1[a]**	**2[b]**	**3[c]**	**4[d]**	**5[e]**	**6[f]**	**7[g]**	**8[h]**	
Shah, N.	2019	yes	yes	yes	yes	yes	no	yes	yes	7good
Uchida, A.	2017	yes	yes	yes	yes	yes	no	yes	yes	7good
Vakil, V.	2018	yes	yes	yes	yes	yes	no	yes	yes	7good
Pichler Sekulic, Simona	2020	yes	yes	yes	yes	yes	no	yes	yes	7good
Tabei, A.	2018	yes	yes	yes	yes	yes	no	yes	yes	7good
Saly, D. L.	2018	yes	yes	yes	no	yes	no	yes	yes	6good
Taki, T.	2020	yes	yes	yes	yes	yes	no	yes	yes	7good
Person, F.	2020	yes	yes	yes	yes	yes	no	yes	yes	7good
Murakami, N.	2016	yes	yes	yes	yes	yes	no	yes	yes	7good
Kawakado, K.	2021	yes	yes	yes	no	yes	no	yes	yes	6good
Glutsch, V.	2019	yes	yes	yes	yes	yes	no	yes	yes	7good
Gallan, A. J.	2019	yes	yes	yes	yes	yes	no	yes	yes	7good
Herrmann, S.	2021	yes	yes	yes	no	yes	no	yes	yes	6good
Rashidi, A.	2021	yes	yes	yes	yes	yes	no	yes	yes	7good
Oki, R.	2020	yes	yes	yes	no	yes	no	no	yes	5fair
Hu, Q.	2020	yes	yes	yes	yes	yes	no	yes	yes	7good
Faieta, A.	2021	yes	yes	yes	yes	yes	no	yes	yes	7good
Okawa, S.	2020	yes	yes	yes	no	yes	no	yes	yes	6good
Venkatachalam, K.	2020	yes	yes	yes	no	no	no	yes	yes	5fair
Bonilla, M.	2021	yes	yes	yes	yes	yes	no	yes	yes	6good

Supplemental Table S6. (continued)

Padala, S. A.	2021	yes	yes	yes	yes	yes	no	yes	yes	7good
Kim, D. W.	2021	yes	yes	yes	yes	yes	no	no	yes	6good
Hayata, Manabu	2020	yes	yes	yes	yes	yes	no	yes	yes	7good
Bickel, A.	2016	yes	yes	yes	no	yes	no	yes	yes	7good
Mukherjee, A.	2020	yes	yes	yes	yes	yes	no	yes	yes	6good
Wakabayashi, K.	2021	yes	yes	yes	no	yes	no	no	yes	6good
Tanabe, K.	2020	yes	yes	yes	yes	yes	no	yes	yes	6good
Nakatani, Y.	2018	yes	yes	yes	yes	yes	no	yes	yes	7good
Daanen, R. A.	2017	yes	yes	yes	yes	yes	no	yes	yes	7good
Shim, J.	2021	yes	yes	yes	no	yes	no	yes	yes	7good
Ai, Luoyan	2021	yes	yes	yes	yes	yes	no	yes	yes	6good
Jung, Kyungsuk	2016	yes	yes	yes	yes	yes	no	yes	yes	7good
Kitchlu, A.	2017	yes	yes	yes	yes	yes	no	yes	yes	6good
Messias, Ana	2019	yes	yes	yes	no	yes	no	no	yes	7good
Vaughan, E.	2020	yes	yes	yes	yes	yes	no	yes	yes	5fair
Gao, B.	2018	yes	yes	yes	yes	yes	no	no	yes	6good
Cruz-Whitley, J.	2020	yes	yes	yes	yes	yes	no	yes	yes	7good
Izzedine, H.	2021	yes	yes	yes	yes	yes	no	yes	yes	7good
Lemoine, M.	2019	yes	yes	yes	yes	yes	no	yes	yes	7good
Kidd, J. M.	2016	yes	yes	yes	no	yes	no	yes	yes	7good
Thajudeen, B.	2015	yes	yes	yes	yes	yes	no	yes	yes	6good
Escandon, J.	2017	yes	yes	yes	no	yes	no	no	yes	7good
de Sousa Oliveira, D.	2019	yes	yes	yes	yes	yes	no	no	yes	4fair

Supplemental Table S6. (continued)

Lin, J. S.	2021	yes	yes	yes	no	yes	no	yes	yes	7good
Parza, K.	2021	yes	yes	yes	yes	yes	no	yes	yes	6good
Lapman, S.	2020	yes	yes	yes	yes	yes	no	yes	yes	7good
Koda, R.	2018	yes	yes	yes	yes	yes	no	yes	yes	7good
Tawhari, I.	2020	yes	yes	yes	yes	yes	no	yes	yes	7good
Takahashi, N.	2018	yes	yes	yes	yes	yes	no	yes	yes	7good
Sise, M. E.	2019	yes	yes	yes	no	yes	no	yes	yes	7good
Gebauer, E.	2021	yes	yes	yes	yes	yes	no	yes	yes	6good
Cortazar, F. B.	2016	yes	yes	yes	yes	yes	no	yes	yes	7good
Aratani, S.	2021	yes	yes	yes	no	yes	no	yes	yes	7good
Mamlouk, O.	2020	yes	yes	yes	yes	yes	no	yes	yes	6good
Chemaly, E. R.	2020	yes	yes	yes	yes	yes	no	yes	yes	7good
Martínez Valenzuela, L.	2021	yes	yes	yes	yes	yes	no	yes	yes	7good
Izzedine, H.	2014	yes	yes	yes	yes	yes	no	yes	yes	7good
Shirali, A. C.	2021	yes	yes	yes	yes	yes	no	yes	yes	7good
Noto-Kadou-Kaza, B.	2021	yes	yes	yes	yes	yes	no	yes	yes	7good
Sammartino, C.	2010	yes	yes	yes	yes	yes	no	yes	yes	7good
Jolly, E. C.	2009	yes	yes	yes	yes	yes	no	yes	yes	7good
Uner, M.	2021	yes	yes	yes	yes	yes	no	yes	yes	7good
Basnet, S.	2019	yes	yes	yes	no	yes	no	yes	yes	7good
Xipell, M.	2018	yes	yes	yes	yes	yes	no	yes	yes	6good
Irifuku, T.	2020	yes	yes	yes	no	yes	no	yes	yes	7good
Gordon, L.	2019	yes	yes	yes	no	yes	no	yes	yes	6good

Supplemental Table S6. (continued)

Ryuzaki, M.	2019	yes	yes	yes	yes	yes	no	yes	yes	6good
Belliere, J.	2016	yes	yes	yes	no	yes	no	yes	yes	7good
Georgianos, P. I.	2019	yes	yes	yes	yes	yes	no	yes	yes	6good
Oleas, Diana	2021	yes	yes	yes	no	yes	no	yes	yes	7good
Mulroy, M.	2021	yes	yes	yes	yes	yes	no	yes	yes	6good
Bottlaender, Lea	2017	yes	yes	yes	yes	yes	no	yes	yes	7good
Buyansky, Dimitry	2017	yes	yes	yes	yes	yes	no	yes	yes	7good
Patel, V.	2020	yes	yes	yes	yes	yes	no	yes	yes	7good
Charmetant, X.	2019	yes	yes	yes	yes	yes	no	no	yes	6good
Mamlouk, O.	2020	yes	yes	yes	no	yes	no	no	yes	5fair
Soellradl, I.	2018	yes	yes	yes	no	yes	no	no	yes	5fair
Toda, M. G.	2021	yes	yes	yes	yes	yes	no	no	yes	5fair
Oliveira, D. S.	2019	yes	yes	yes	yes	yes	no	yes	yes	6good
Tanaka, A.	2017	yes	yes	yes	no	yes	no	yes	yes	7good
M. Espi	2020	yes	yes	yes	yes	yes	no	yes	yes	6good
Sebastian Hultin	2020	yes	yes	yes	yes	yes	no	no	yes	6good
Omar Mamlouk	2019	yes	yes	yes	no	yes	no	no	yes	5fair
Alejandro Meraz- Muñoz	2020	yes	yes	yes	no	yes	no	yes	yes	6good
Okamoto, M.	2020	yes	yes	yes	yes	yes	no	no	yes	6good
Narayanankutty, N. P.	2019	yes	yes	yes	yes	yes	no	no	yes	6good
Marvania, N.	2020	yes	yes	yes	yes	yes	no	yes	yes	7good
Catapano, F.	2021	yes	yes	yes	yes	yes	no	yes	yes	7good
Panthofer, A. M.	2020	yes	yes	yes	yes	yes	no	yes	yes	7good

Supplemental Table S6. (continued)

Chua, J.	2020	yes	yes	yes	yes	yes	no	yes	yes	7good
George, S.	2019	yes	yes	yes	yes	yes	no	yes	yes	7good
Mamlouk, O.	2019	yes	yes	yes	yes	yes	no	yes	yes	7good
Pampols, P. A.	2020	yes	yes	yes	yes	yes	no	yes	yes	7good
Abramson, M.	2020	yes	yes	yes	yes	yes	no	yes	yes	7good
Lo, W. K.	2020	yes	yes	yes	yes	yes	no	yes	yes	7good
Leuprecht, L.	2020	yes	yes	yes	yes	yes	no	yes	yes	7good
Bobart, S.	2020	yes	yes	yes	yes	yes	no	yes	yes	7good
Mohan, S.	2018	yes	yes	yes	yes	yes	no	yes	yes	7good
Savedchuk, S.	2019	yes	yes	yes	yes	yes	no	yes	yes	7good
Tadros, M. G.	2019	yes	yes	yes	yes	yes	no	yes	yes	7good
Montanez, M.	2020	yes	yes	yes	yes	yes	no	yes	yes	7good
Lin, J.	2020	yes	yes	yes	yes	yes	no	yes	yes	7good
Duque, J.	2018	yes	yes	yes	yes	yes	no	yes	yes	7good
Baradhi, K. M.	2018	yes	yes	yes	yes	yes	no	yes	yes	7good
Leger, K.	2019	yes	yes	yes	yes	yes	no	yes	yes	7good
Nagaraju, G.	2018	yes	yes	yes	yes	yes	no	yes	yes	7good
Jensen, C.	2019	yes	yes	yes	yes	yes	no	no	yes	6good
Meraz-Munoz, A. Y.	2019	yes	yes	yes	yes	yes	no	no	yes	6good
Makati, D.	2018	yes	yes	yes	yes	yes	no	yes	yes	7good
Hninn, W. Y.	2019	yes	yes	yes	yes	yes	no	yes	yes	7good
Leonard, D.	2020	yes	yes	yes	yes	yes	no	yes	yes	7good
Annamaraju, P.	2020	yes	yes	yes	yes	yes	no	yes	yes	7good

Supplemental Table S6. (continued)

Hassanein, M.	2020	yes	yes	yes	no	yes	no	yes	yes	6good
Karo, N. L.	2020	yes	yes	yes	no	yes	no	yes	yes	6good
Thomas, M. J. A.	2018	yes	yes	yes	yes	yes	no	yes	yes	7good
Efe, O.	2020	yes	yes	yes	no	yes	no	yes	yes	6good
Chang, Y.	2020	yes	yes	yes	yes	yes	no	yes	yes	7good
Sebastian, A. D.	2018	yes	yes	yes	yes	yes	no	no	yes	6good
Vishwanath, M.	2018	yes	yes	yes	yes	yes	no	yes	yes	7good
Molteni, A.	2018	yes	yes	yes	yes	yes	no	yes	yes	7good
Fadel, N.	2009	yes	yes	yes	yes	yes	no	yes	yes	7good

a. Did the researcher(s) specified why this case was reported?

b. Was the exposure adequately ascertained?

c. Was the outcome adequately ascertained?

d. If other alternative causes(any other medication except NSAIDS/PPI that was possible to cause renal lesions) was reported, were they ruled out?

e. Was there a challenge/rechallenge phenomenon, or was renal function stable after cessation of ICI/corticosteroid treatment?

f. Was there a dose–response effect?

g. Was follow-up long enough for renal outcomes or death to occur?

h. Is the case(s) described with sufficient details to allow other investigators to replicate the research or to allow practitioners make inferences related to their own practice?

Supplemental Table S7. Risk factors for ICI-associated kidney IRAEs with ATIN/AIN and ATN

		Univariate logistic analysis		Multiple logistic analysis			Univariate logistic analysis	
	ATIN/AIN (n=143)	P value	OR (95 CI)	P value	OR (95 CI)	ATN(n=11)	P value	OR (95 CI)
**Age**	68(62,74)	**0.001**	1.040(1.015,1.065)	**0.006**	1.055(1.016,1.097)	63(58,70)	0.402	0.982(0.940,1.025)
**Male**	92/143(64.3)	**0.034**	0.503(0.266,0.951)	**0.026**	0.321(0.118,0.875)	11/11(100)	0.997	
**Comorbidities**								
**Hypertension**	55/107(51.4)	0.273	1.461(0.742,2.876)			5/10(50.0)	0.917	1.070(0.297,3.854)
**Diabetics**	15/108(13.9)	0.135	0.524(0.225,1.222)			2/10(20.0)	0.793	1.240(0.248,6.191)
**CKD**	27/108(25.0)	0.742	0.881(0.415,1.872)			3/10(30.0)	0.753	1.252(0.308,5.086)
**CHD**	9/108(8.3)	0.492	0.682(0.229,2.031)			2/10(20.0)	0.254	2.615(0.502,13.627)
**Tumor type**		**0.014**		0.446			0.934	
**Melanoma**	62/143(43.4)					0/11(0)		
**Lung cancer**	42/143(29.4)	0.625	1.210(0.564,2.593)	0.092		4/11(36.4)	0.997	
**Renal cancer**	11/143(7.7)	0.102	0.444(0.167,1.175)	0.783		1/11(9.1)	0.997	
	7/143(4.9)	**0.004**	0.339(0.161,0.712)	0.868		1/11(9.1)	0.997	
**Hematological cancer**								
**Others**	21/143(14.7)	0.290	2.083(0.535,8.106)	0.170		5/11(45.5)	0.996	
**ICI type**		0.642					0.999	
**PD-1**	93/137(67.9)					9/10(90.0)		
**PD-L1**	6/137(4.4)	0.448	0.619(0.180,2.134)			1/10(10.0)	0.999	

Supplemental Table S7. (continued)

**CTLA-4**	9/137(6.6)	0.443	0.664(0.233,1.891)			0/10(0)	0.998	
**Combination**	29/137(21.2)	0.568	1.247(0.585,2.661)			0/10(0)	0.729	
**Only ICI**	18/121(14.9)	0.814	0.898(0.366,2.203)			7/11(63.6)	**0.045**	0.262(0.071,0.968)
**PPI**	44/98(44.9)	**0.016**	2.954(1.227,7.108)	**0.010**	3.620(1.357,9.658)	2/7(28.6)	0.582	0.624(0.117,3.341)
**NSAIDs**	24/98(24.5)	**0.044**	3.676(1.035,13.050)	0.105		0/7(0)	0.998	<0.001
**Baseline Scr level**	1.0(0.8,1.2)	0.541	1.306(0.555,3.072)			0.80(0.63,1.18)	0.512	0.498(0.062,4.017)
**Extra renal irAEs**	46/130(35.4)	**0.082**	1.765(0.931,3.345)	0.452		3/11(30.0)	0.940	0.948(0.237,3.792)

Abbreviations: IRAE: immune related adverse events; ICI: immune checkpoint inhibitor; CKD: chronic kidney disease; CHD: coronary heart disease; PPI: proton pump inhibiter; NSAIDs: non-steroidal anti-inflammatory drugs.

Supplemental Table S8. Risk factors for ICI-associated kidney IRAEs with glomerular disease and systematic disease

		**Univariate logistic analysis**		**Multiple logistic analysis**			**Univariate logistic analysis**	
	**Glomerular (n=46)**	**P value**	**OR (95 CI)**	**P value**	**OR (95 CI)**	**Systematic disease (n = 39)**	**P value**	**OR (95 CI)**
**Age**	61(46,69)	**<0.001**	0.954(0.930,0.979)	**0.001**	0.933(0.895,0.973)	65(55,71)	0.518	0.991(0.965,1.018)
**Male**	35/46(76.1)	0.260	1.537(0.728,3.246)			28/39(71.8)	0.702	1.161(0.540,2.493)
**Comorbidities**								
**Hypertension**	13/30(43.3)	0.537	0.777(0.349,1.732)			9/24(37.5)	0.249	0.591(0.242,1.444)
**Diabetics**	7/30(23.3)	0.307	1.659(0.628,4.381)			3/25(12.0)	0.473	0.625(0.173,2.258)
**CKD**	6/30(20.0)	0.423	0.571(0.253,1.780)			9/25(36.0)	0.208	1.793(0.723,4.446)
**CHD**	3/30(10.0)	0.906	1.083(0.286,5.107)			2/25(8.0)	0.790	0.809(0.171,3.829)
**Tumor type**		**<0.001**		0.052			0.508	
**Melanoma**	8/46(17.4)					20/39(51.3)		
**Lung cancer**	6/46(13.0)	0.766	1.185(0.388,3.618)	0.181		6/39(15.4)	0.069	0.402(0.150,1.074)
**Renal cancer**	9/46(19.6)	**0.001**	7.406(2.394,22.916)	0.042		4/39(10.3)	0.697	0.788(0.238,2.612)
	7/46(15.2)	<0.001	17.281(4.146,72.038)	0.086		0/39(0)	0.999	<0.001
**Hematological cancer**								
**Others**	16/46(34.8)	**0.001**	5.267(2.043,13.578)	0.517		9/39(23.1)	0/650	0.815(0.337,1.971)
**ICI type**		0.771						

Supplemental Table S8. (continued)

**PD-1**	31/42(73.8)				22/37(59.5)	0.488	
**PD-L1**	2/42(4.8)	0.931	0.926(0.163,5.251)		2/37(5.4)	0.921	1.091(0.196,6.067)
**CTLA-4**	2/42(4.8)	0.769	1.268(0.260,6.176)		5/37(13.5)	0.821	0.832(0.168,4.113)
**Combination**	7/42(16.7)	0.685	0.643(0.076,5.417)		8/37(21.6)	0.451	2.045(0.318,13.159)
**Only ICI**	33/34(97.1)	**0.065**	6.818(0.891,52.201)	0.060	25/29(86.2)	0.913	1.066(0.338,3.360)
**PPI**	6/26(23.1)	**0.078**	0.411(0.153,1.104)	0.275	3/16(81.3)	**0.096**	0.330(0.089,1.219)
**NSAIDs**	5/26(19.2)	0.913	0.942(0.319,2.777)		2/16(87.5)	0.431	0.537(0.114,2.520)
**Baseline Scr level**	0.82(0.76,1.18)	0.423	0.646(0.222,1.878)		1.00(0.80,1.32)	0.679	1.229(0.464,3.254)
**Extra renal irAEs**	8/46(17.4)	**0.026**	0.391(0.171,0.896)	0.200	10/38(26.3)	0.484	0.754(0.342,1.663)

Abbreviations: IRAE: immune related adverse events; ICI: immune checkpoint inhibitor; CKD: chronic kidney disease; CHD: coronary heart disease; PPI: proton pump inhibiter; NSAIDs: non-steroidal anti-inflammatory drugs.

Supplemental Table S9. Univariate and multivariable logistic analysis of factors with kidney function recovery in AKD patients

	Univariate logistic analysis				Multivariable logistic analysis	
	**No recovery (n=31)**	**Renal recovery (n=137)**	**P value**	**OR (95%CI)**	**P value**	**OR (95%IC)**
**Age**	63(52,69)	68(62,74)	**0.012**	1.044(1.010,1.080)	**0.051**	1.046 (1.000,1.095)
**Male [n/N(%)]**	25/31(80.6)	89(65.0)	**0.097**	0.445(0.171,1.159)	0.538	
**Comorbidities**						
**Hypertension**	6/19(31.6)	49/95(51.6)	0.118	2.308(0.810,6.580)		
**Diabetics**	1/19(5.3)	21/95(22.1)	0.123	5.108(0.644,40.528)		
**CKD**	6/19(31.6)	20/95(21.1)	0.322	0.578(0.195,1.711)		
**CHD**	1/19(5.3)	6/95(6.3)	0.862	1.213(0.138,10.700)		
**Tumor type**			0.471			
**Melanoma**	14/31(45.2)	46/137(33.6)				
**Lung cancer**	5/31(16.1)	44/137(32.1)	0.080	2.678(0.890,8.059)		
**Renal cancer**	3/31(9.7)	16/137(11.7)	0.489	1.623(0.412,6.391)		
**Hematological cancer**	1/31(3.2)	4/137(2.9)	0.865	1.217(0.126,11.801)		
**Others**	8/31(25.8)	27/137(19.7)	0.958	1.027(0.382,2.765)		
**ICI type**			0.104			
**PD-1**	20/31(64.5)	97/137(70.8)				
**PD-L1**	2/31(6.5)	7/137(5.1)	0.697	0.722(0.139,3.733)		
**CTLA-4**	6/31(19.4)	8/137(5.8)	0.030	0.275(0.086,0.879)		

Supplemental Table S9. (continued)

**Combination**	3/31(9.7)	25/137(18.2)	0.411	1.718(0.473,6.246)		
**Only ICI**	21/24(87.5)	87/108(80.6)	0.429	0.592(0.161,2.172)		
**Interval time(d)**	147(30,267)	106(60,180)	0.523	0.999(0.997,1.001)		
**Baseline Scr**	1.02(0.85,1.22)	1.00(0.80,1.18)	0.487	0.650(0.193,2.189)		
**ATIN/AIN**	12/31(38.7)	104/137(75.9)	**<0.001**	4.990(2.193,11.352)	0.855	
**ATN**	1/31(3.2)	7/137(5.1)	0.659	1.615(0.191,13.628)		
**Glomerular disease**	3/31(9/7)	24/137(17.5)	0.291	1.982（0.557，7.055）		
**Systematic disease**	17/31(54.8)	11/137(8.0)	**<0.001**	0.072(0.028,0.184)	**<0.001**	0.119 (0.038,0.376)
**Extra-renal irAE**	7/30(23.3)	44/135(32.6)	0.324	1.589(0.633,3.984)		
**AKD grade**			**0.051**	1.689(0.999,2.856)	0.177	
**AKD 1 grade**	7/30(23.3)	11/129(8.5)				
**AKD 2 grade**	5/30(16.7)	24/129(18.6)				
**AKD 3 grade**	18/30(60.0)	94/129(72.9)				
**corticosteroid**	25/31(80.6)	131/137(95.6)	**0.007**	5.240(1.563,17.568)	**0.007**	9.429 (1.823,48.779)
**Intravenous corticosteroid**	11/28(39.3)	44/131(33.6)	0.566	0.782(0.337,1.812)		
**Pulse steroid therapy**	7/8(87.5)	23/33(69.7)	0.326	0.329(0.036,3.034)		
**Oral corticosteroid**			0.349	1.611(0.594,4.370)		
**Low-dose (<0.5)**	4/16(25.0)	13/100(13.0)				

Supplemental Table S9. (continued)

**Moderate-dose (0.5-1.0)**	10/16(62.5)	73/100(73.0)				
**High-dose (>1.0)**	2/16(12.5)	14/100(14.0)				
**immunosuppressants**	9/28(32.1)	25/133(18.8)	0.121	0.489(0.198,1.207)		
**Discontinued ICI**	21/25(84.0)	121/125(96.8)	**0.019**	5.762(1.336,24.843)		
**RRT**	17/27(63.0)	17/135(12.6)	**0.001**	0.085(0.033,0.215)	**<0.001**	0.111 (0.033,0.374)

Abbreviations: IRAE: immune related adverse events; ICI: immune checkpoint inhibitor; CKD: chronic kidney disease; CHD: coronary heart disease; PPI: proton pump inhibiter; NSAIDs: non-steroidal anti-inflammatory drugs; SCr: serum creatinine; AKD: acute kidney disease; RRT: renal replacement treatment.

Supplemental Table S10. Univariate and multivariable logistic analysis of factors with tumor progression and death

		**Univariate logistic analysis**		**Multiple logistic analysis**			**Univariate logistic analysis**		**Multiple logistic analysis**	
	**PD (n=41)**	**P value**	**OR(95%CI)**	**P value**	**OR(95%CI)**	**Death (n=28)**	**P value**	**OR(95%CI)**	**P value**	**OR(95%CI)**
**Age**	64(55,73)	0.184	0.978(0.947,1.011)			64(53,72)	0.399	0.987(0.957,1.018)		
**Male [n/N(%)]**	30/41(73.2)	0.992	1.005(0.399,2.530)			24/28(85.7)	**0.045**	3.136(1.026,9.583)	0.213	
**Comorbidities**										
**Hypertension**	16/35(45.7)	0.376	0.667(0.272,1.636)			11/22(50.0)	0.883	1.073(0.420,2.741)		
**Diabetics**	6/35(17.1)	0.673	0.782(0.249,2.458)			6/22(27.3)	0.542	1.396(0.477,4.081)		
**CKD**	9/35(25.7)	0.828	0.894(0.326,2.453)			8/22(36.4)	0.144	2.217(0.773,5.855)		
**CHD**	2/35(5.7)	0.833	1.242(0.166,9.299)			2/22(9.1)	0.747	1.317(0.247,7.021)		
**Tumor type**		0.464					**0.063**		**0.051**	
**Melanoma**	10/41(24.4)					10/28(35.7)				
**Lung cancer**	10/41(24.4)	0.592	1.357(0.445,4.142)			2/28(7.1)	0.037	0.186(0.039,0.902)	0.082	0.230(0.044,1.202)
**Renal cancer**	6/41(14.6)	0.595	1.425(0.386,5.262)			6/28(21.4)	0.349	1.757(0.540,5.719)	0.674	1.343(0.339,5.321)
**Hematological cancer**	5/41(12.2)	0.092	4.750(0.778,29.019)			3/28(10.7)	0.182	3.075(0.591,15.993)	**0.047**	13.342(1.032,172.512)
**Others**	10/41(24.4)	0.215	2.111(0.647,6.885)			7/28(25.0)	0.888	0.926(0.317,2.706)	0.359	0.547(0.151,1.984)
**ICI type**		0.621					0.658			
**PD-1**	31/41(75.6)					19/28(67.9)				

Supplemental Table S10. (continued)

**PD-L1**	4/41(9.8)	0.291	2.581(0.444,15.015)			2/28(7.1)	0.526	1.719(0.322,9.170)		
**CTLA-4**	2/41(4.9)	0.804	1.290(0.172,9.681)			3/28(10.7)	0.280	2.211(0.524,9.320)		
**Combination**	4/41(9.8)	0.505	0.645(0.178,2.340)			4/28(14.3)	0.856	0.897(0.278,2.890)		
**Only ICI**	30/39(76.9)	0.313	0.569(0.191,1.170)			21/25(84.0)	0.984	1.012(0.306,3.346)		
**Interval time(d)**	102(60,180)	0.647	0.999(0.996,1.003)			68(33,125)	0.197	0.998(0.995,1.001)		
**Baseline Scr**	0.90(0.79,1.30)	0.743	1.178(0.443,3.137)			1.01(0.80,1.40)	0.360	1.735(0.533,5.649)		
**ATIN/AIN**	19/41(46.3)	**0.099**	0.497(0.216,1.145)	0.405		7/28(25.0)	**<0.001**	0.180(0.071,0.455)	**0.001**	0.164(0.057,0.473)
**ATN**	4/41(9.8)	0.474	1.766(0.372,8.375)			3/28(10.7)	0.283	2.177(0.527,8.997)		
**Glomerular disease**	17/41(41.5)	**0.021**	2.975(1.176,7.527)	0.021	2.975(1.176,7.527)	9/28(32.1)	0.275	1.642(0.674,4.003)		
**Systematic disease**	6/41(14.6)	0.223	0.514(0.176,1.499)			11/28(39.3)	**0.011**	3.123(1.293,7.539)	0.335	
**Extra-renal irAE**	9/40(22.5)	0.553	0.747(0.284，1.960)			6/28(21.4)	0.498	0.712(0.267,1.899)		
**AKD grade**		0.303	0.730(0.402,1.328)				0.739	1.114(0.590,2.105)		
**AKD 1 grade**	7/37(18.9)					4/26(15.4)				
**AKD 2 grade**	7/37(18.9)					2/26(7.1)				
**AKD 3 grade**	23/37(62.2)					20/26(76.9)				
**corticosteroid**	37/41(90.2)	0.265	0.370(0.064,2.129)			26/28(92.9)	0.954	1.048(0.217,5.069)		
**Intravenous corticosteroid**	10/38(26.3)	0.278	0.602(0.240,1.507)			8/27(29.6)	0.950	0.972(0.392,2.408)		
**Pulse steroid therapy**	5/7(71.4)	0.858	0.833(0.114,6.111)			7/8(87.5)	0.301	3.294(0.345,31.490)		

Supplemental Table S10. (continued)

**Oral corticosteroid**		0.420	1.495(0.562,3.974)		0.229		
**Low-dose (<0.5)**	2/30(6.7)			1/22(4.5)			
**Moderate-dose (0.5-1.0)**	23/30(76.7)			16/22(72.7)	0.108	0.150(0.015,1.518)	
**High-dose (>1.0)**	5/30(16.7)			5/22(22.7)	0.219	0.465(0.137,1.579)	
**immunosuppressants**	12/41(29.3)	0.677	0.828(0.340,2.015)	7/27(25.9)	0.705	1.202(0.464,3.113)	
**Corticosteroid or immunosuppressants**	37/41(90.2)	0.134	5.514(0.592,51.368)	26/28(92.9)	0.935	1.068(0.218,5.236)	
**Discontinued ICI**	36/37(97.3)	0.289	0.299(0.032,2.793)	26/28(92.9)	0.596	1.564(0.299,8.188)	
**RRT**	5/38(13.2)	0.331	0.565(0.178,1.788)	8/27(29.6)	**0.091**	2.266(0.878,5.848)	0.339
**No recovery of kidney function**	6/31(80.6)	0.487	1.584(0.434,5.787)	8/24(33.3)	**0.005**	4.455(1.554,12.767)	0.053

Abbreviations: IRAE: immune related adverse events; ICI: immune checkpoint inhibitor; CKD: chronic kidney disease; CHD: coronary heart disease; SCr: serum creatinine; AKD: acute kidney disease; RRT: renal replacement treatment.
